# Extensive Bone Marrow Necrosis and Osteolytic Lesions in a Case of Acute Myeloid Leukemia Transformed from Polycythemia Vera

**DOI:** 10.7759/cureus.639

**Published:** 2016-06-13

**Authors:** Isaac Chambers, Phu Truong, K. James Kallail, William Palko

**Affiliations:** 1 Internal Medicine, University of Kansas School of Medicine-Wichita; 2 Wesley Medical Center, University of Kansas School of Medicine-Wichita

**Keywords:** marrow, necrosis, osteolytic, granulocytic, sarcoma, polycythemia, myeloid, leukemia, myeloma, myelofibrosis

## Abstract

Acute myeloid leukemia (AML) is the most common leukemia in adults. In rare cases, bone marrow necrosis (BMN) and osteolytic lesions are presenting features of AML. The following case describes a patient with known polycythemia vera (PV) that presented with signs of multiple myeloma, including hypercalcemia, anemia, and lytic lesions of the thoracic spine and skull. Laboratory workup was not indicative of myeloma. A bone marrow biopsy was performed, which revealed extensive BMN and initial pathology was consistent with metastatic carcinoma. However, no immunohistochemical stains could be performed due to the extent of BMN; a repeat biopsy was therefore performed. Flow cytometry and CD45 staining were consistent with PV that had transformed to AML. Due to the patient’s comorbidities, she was a poor candidate for stem cell transplant and did not wish to pursue chemotherapy. Ultimately, she pursued hospice care. Based on our literature review, both BMN and osteolytic lesions are rare manifestations of AML and have not been reported to occur simultaneously. These findings can lead to a diagnostic dilemma and suspicion of other malignancies. This case demonstrates that AML should remain in the differential diagnosis in those patients who present with BMN and osteolytic lesions.

## Introduction

Acute myeloid leukemia (AML) is the most common leukemia in adults [1]. Acute leukemia can develop from myeloproliferative neoplasms, including polycythemia vera (PV). PV is estimated to transform into acute leukemia in 5-15% of cases over the course of 10 years [2-3]. AML leads to cytopenias and clinically presents with fatigue, dyspnea, infection, and bleeding. In rare cases, AML can present with bone marrow necrosis (BMN) [4] or granulocytic sarcoma (GS) [5].

BMN is a histopathologic diagnosis characterized by destruction of the medullary stroma with preservation of cortical bone. BMN often presents with fatigue and acute onset of bone pain. Laboratory abnormalities can include anemia, leukopenia, thrombocytopenia, elevated lactate dehydrogenase, and elevated alkaline phosphatase. The degree of necrosis is defined as mild, moderate, or severe if necrosis in the biopsy sample is less than 20%, 20 to 50%, or greater than 50%, respectively. Severe BMN occurs in about 2.4% of AML [4].

Granulocytic sarcoma (GS), also referred to as chloroma, myeloblastoma, and myelosarcoma, is an extramedullary tumor of myeloid cells that most often presents in soft tissue, bones, periosteum, and lymph nodes. GS incidence is highest in certain types of leukemia, such as infantile leukemia and AML with t(8;21). Of adult leukemias, the FAB M4/5 types are associated with an increased incidence of GS. In a review of case reports, GS was found simultaneously with AML in 14 of 32 cases, predated leukemia by 0.5 - 24 months in five cases, and was detected after the diagnosis by 5 - 60 months in 14 cases. Overall, the incidence of GS in AML is 3-5% [5].

The following case describes a patient with known PV that transformed to AML who presented with both osteolytic lesions and bone marrow necrosis.

## Case presentation

A 62-year-old female with a history of PV and four years of hydroxyurea treatment presented to an outside hospital for increasing weakness, confusion, and a fall. Her calcium level at the outside hospital was 11.3 mg/dL with a hemoglobin level of 6.9 g/dL. A CT scan of the chest, abdomen, and pelvis revealed a lytic lesion around the lateral aspect of the T2 vertebral body.

The patient initially had improvement of her cognitive status with intravenous fluids, bicarbonate, and bisphosphonate therapy but later became more disoriented, thus leading to the transfer to our medical center. On admission to our facility, the patient was overall doing fair with a mildly elevated heart rate but otherwise normal vital signs. Her GCS was 14 on admission due to confused speech. She was otherwise was alert, oriented, and following commands. The patient verbally consented for diagnostic evaluation and treatment. A complete blood count (CBC) revealed a white blood cell count of 6.1 x 10^6^ ml, hemoglobin level of 7.3 g/dL, mean corpuscular volume of 91.4, and a platelet count of 46 x 10^3^ mL. The CBC differential revealed 1% nucleated RBCs, 0.2% reticulocytes, and 1% schistocytes, with teardrop cells and ovalocytes noted in the specimen. The PTH level was 10 pg/mL and 25-OH vitamin D level was 4.2 ng/mL, thus, ruling out primary hyperparathyroidism and vitamin D toxicity.

**Figure 1:**
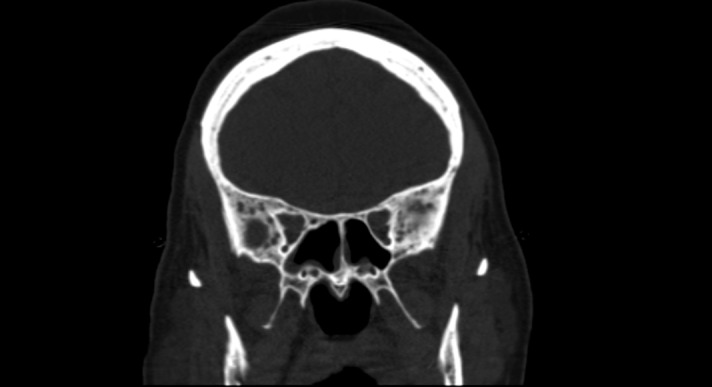
AP view of the skull on CT scan revealing a diffuse heterogeneous appearance of the marrow with scattered lytic lesions.

**Figure 2:**
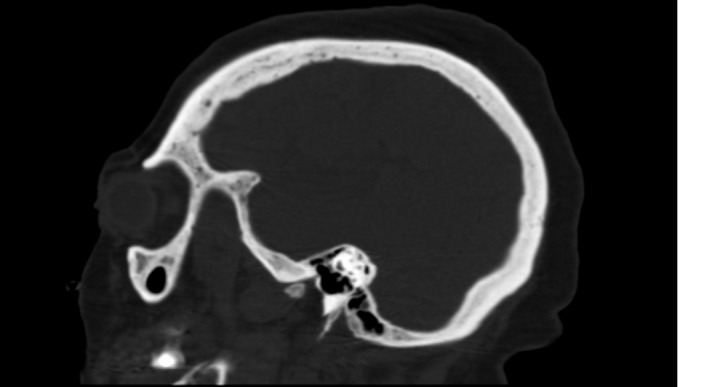
Sagittal view of the skull on CT scan revealing a diffuse heterogeneous appearance of the marrow with scattered lytic lesions.

A CT scan of the head and a bone survey revealed a heterogeneous appearance of the skull with small lytic areas (Figures 1-2). Other images obtained in the bone survey were of poor quality due to the patient's body habitus. Antibodies to human T-cell lymphotropic virus type I and II were negative. A workup for multiple myeloma was pursued. Free kappa light chains were 23 mg/L and free lambda light chains were 35 mg/L, resulting in a ratio of 1.5. Serum and urine electrophoresis showed no evidence of monoclonal gammopathy.

**Figure 3:**
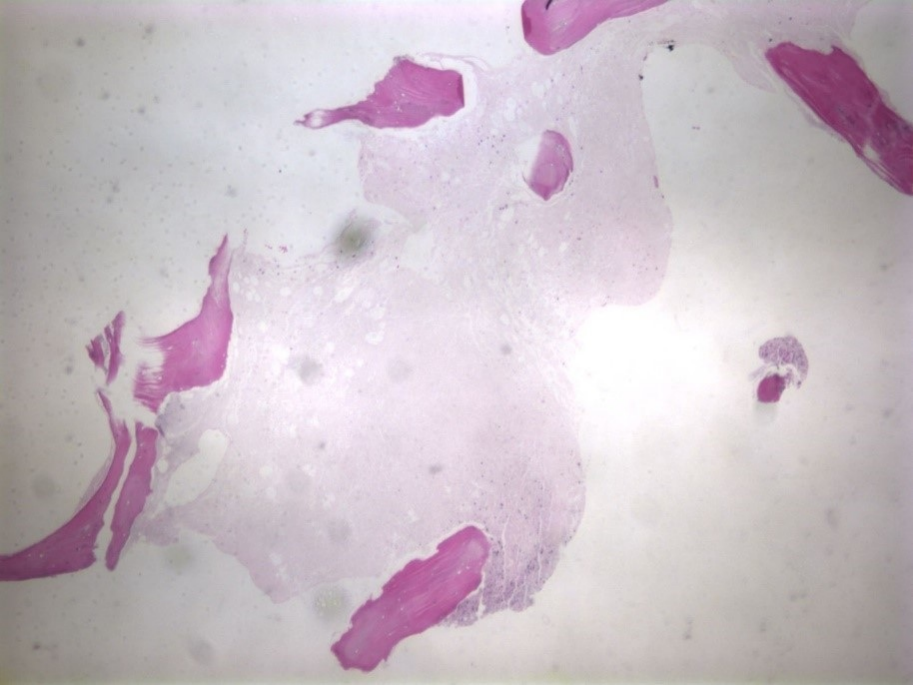
Biopsy of the iliac crest revealing extensive bone marrow necrosis (H&E, 4X magnification).

**Figure 4:**
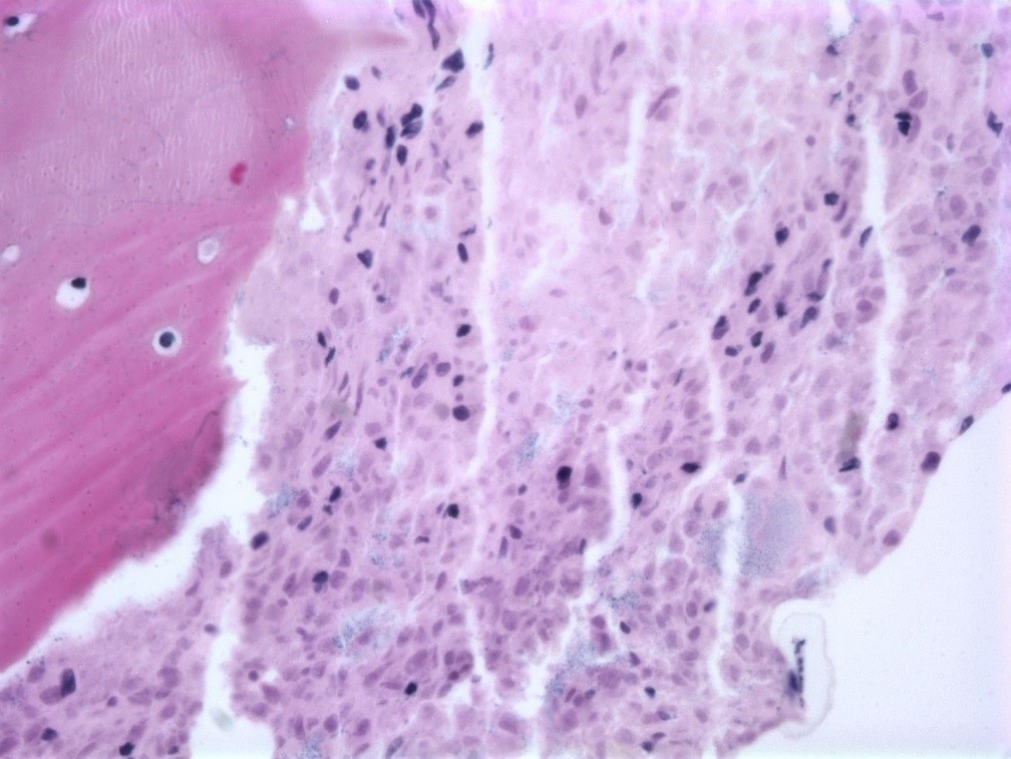
Biopsy of iliac crest revealing extensive bone marrow necrosis (H&E, 40X magnification).

**Figure 5:**
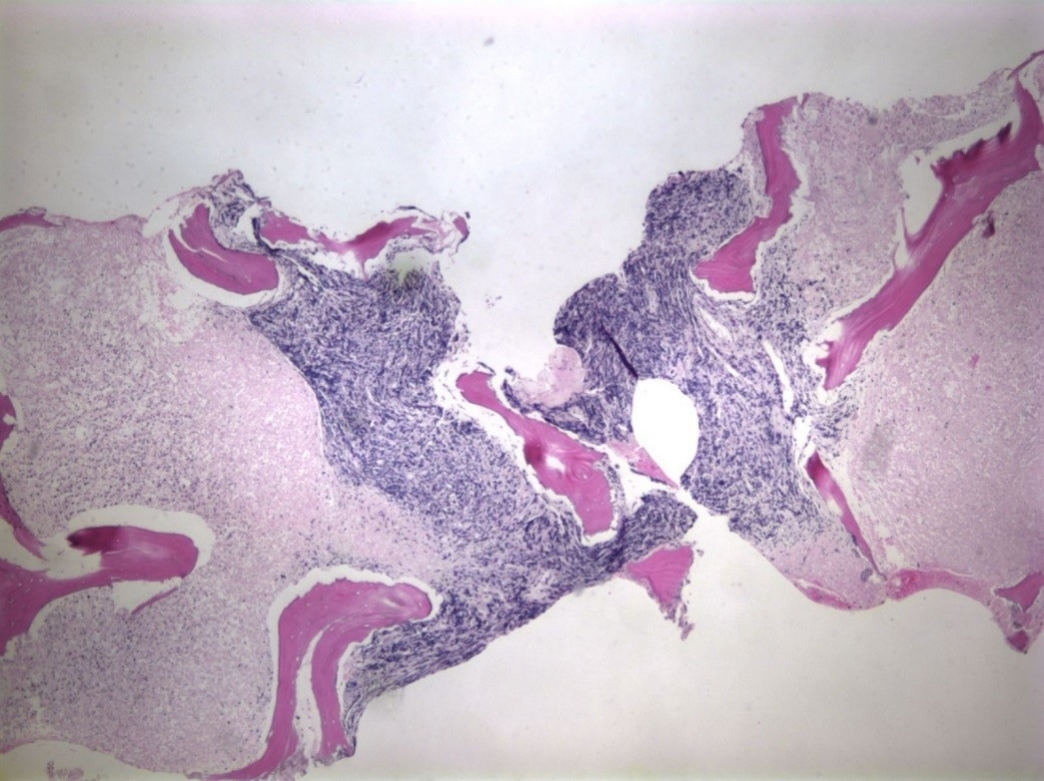
Biopsy of the T2 vertebral body revealing bone marrow necrosis and a focus of viable tumor cells (H&E, 4X magnification).

**Figure 6:**
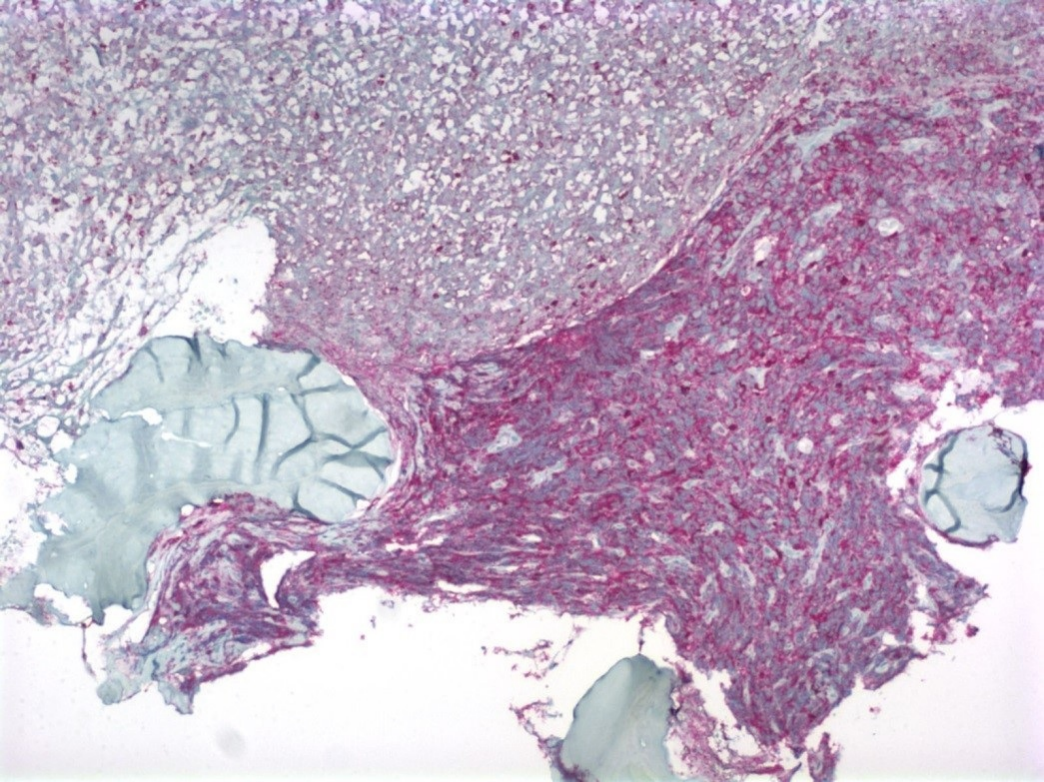
Biopsy of the T2 vertebral body revealing bone marrow necrosis and a focus of viable tumor cells (CD45 staining, 10X magnification).

A bone marrow biopsy of the left iliac bone revealed extensive (99%) necrosis with flow cytometry positive for EpCam, CD138, and CD56, consistent with metastatic carcinoma. No immunohistochemical staining could be performed due to the extent of the necrosis (Figures 3-4) so a repeat biopsy of the T2 vertebral body was performed and sent to the Mayo Clinic (Figure 5). Immunohistochemistry revealed positive staining for CD45 (Figure 6) in support of hematolymphoid malignancy and favoring a myeloid lineage based on CD33 staining. There was no evidence of metastatic carcinoma contrary to the initial bone marrow biopsy report. In addition, the CBC differential revealed peripheral blasts increasing to 6%. The increasing blast count and flow cytometry results on the second biopsy were consistent with polycythemia vera transformation to AML.

The patient’s confusion improved with the treatment of the hypercalcemia with intravenous bisphosphonate and intravenous fluids. Anemia and thrombocytopenia were treated with packed red blood cells and platelet transfusions, respectively. After the pathology was confirmed on the second bone marrow biopsy, the patient was informed about therapeutic options. Due to the patient’s multiple comorbidities, including hypertension, Type 2 diabetes mellitus, morbid obesity, and previous acute ischemic stroke, she was determined to be a poor candidate for stem cell transplant. The patient was given the option of 7 + 3 chemotherapy with cytarabine and doxorubicin as well as palliative care. Given the potential toxicities and related mortality with the chemotherapy, the patient chose to pursue palliative care measures only and was transferred to an inpatient hospice unit.

## Discussion

Bone marrow necrosis is a rare finding that can be secondary to a multitude of etiologies, including hematologic malignancies. The pathogenesis of leukemia leading to BMN is multifactorial and thought to include locally acting cytokines that inhibit bone marrow hematopoiesis [3] and immune complex deposition leading to microvascular compromise [2]. However, the underlying pathophysiology remains poorly understood. Although the presence of BMN has important prognostic implications, it is an underreported entity. The median overall survival of AML with BMN is 3.7 months compared to 14 months in those without BMN [6].

With the exception of multiple myeloma, lytic lesions are rarely associated with hematologic malignancies [7]. Similar to BMN, the pathogenesis of GS is uncertain. It is theorized that tumor-mediated invasion of bone may be facilitated by cell adhesion molecules, such as CD 15, CD 16, and CD 56 [5, 8]. GS is associated with AML in 3-5% of cases; however, when discovered, it is diagnostic of AML regardless of the blast count in the peripheral blood or bone marrow [9]. The finding of GS is a poor prognostic indicator. Overall survival is significantly decreased after the development of GS, although some patients have survived for months and even years. Prognosis is especially poor when GS or medullary recurrence is found following allogeneic bone marrow transplant, with a median survival of less than two months [8].

Our case had many interesting features. The initial presentation was suggestive of multiple myeloma. After myeloma was excluded, the case remained a diagnostic dilemma due to the considerable extent of necrosis and lack of other AML features. It was not until the increase of peripheral blasts was observed, along with the immunohistochemistry and flow cytometry from the second biopsy, that the diagnosis of AML became evident. In this case, PV transformed to AML, which resulted in BMN. This has rarely been described in previous cases [2-3]. It is also very rare for osteolytic lesions to occur with AML. Osteolytic lesions have been described with leukemic transformation in myelofibrosis [10] but, to our knowledge, there has not been a  reported case of AML that has presented with both osteolytic lesions and BMN.

## Conclusions

The association of BMN and osteolytic lesions with AML is rare. The presentation of either of these features can lead to a diagnostic dilemma; lytic lesions may lead to suspicion of other malignancies and BMN makes histopathologic diagnosis more difficult. If the diagnosis remains uncertain, a repeat biopsy should be pursued as GS and BMN have important prognostic implications.
